# New additions to *Sympoventuriaceae* (*Venturiales*, *Dothideomycetes*): three new species from China

**DOI:** 10.3897/mycokeys.133.197363

**Published:** 2026-06-05

**Authors:** Ming-Yi Zhang, Heng Pan, Yu-Lian Ren, Yun-Jie Wu, Zhi-Qing Fang, Bing‑Da Sun, Gang Tao, Zhi-Yuan Zhang

**Affiliations:** 1 College of Eco-Environmental Engineering, Guizhou Minzu University, Guiyang 550025, China College of Eco-Environmental Engineering, Guizhou Minzu University Guiyang China; 2 College of Ecology and Environment (College of Wetland), Southwest Forestry University, Kunming 650224, China College of Ecology and Environment (College of Wetland), Southwest Forestry University Kunming China; 3 China General Microbiological Culture Collection Center, Institute of Microbiology, Chinese Academy of Sciences, Beijing 100101, China China General Microbiological Culture Collection Center, Institute of Microbiology, Chinese Academy of Sciences Beijing China

**Keywords:** Molecular systematics, soil fungi, taxonomy, three new species

## Abstract

Fungi in urban soils are an indispensable component of urban ecosystems, playing a central role in maintaining soil health, promoting nutrient cycling, facilitating plant growth, and safeguarding ecosystem functions. However, taxonomic studies of fungi in urban soils remain extremely limited. During an investigation of culturable fungi from urban soils in China, eight isolates belonging to *Sympoventuriaceae* were isolated. Although recent taxonomic refinements at the generic level within *Sympoventuriaceae* have progressed, the taxonomic status of some monotypic genera remains to be stabilized. Based on integrated morphological characterization and multigene phylogenetic analyses of five loci (ITS, LSU, *tef1*, *tub2*, and *rpb2*), three novel species are described and illustrated: *Scolecobasidium campestre*, *Verruconis obovatus*, and *Veronaeopsis sinensis*. This study provides comprehensive morphological descriptions, illustrations, phylogenetic data, and comparisons with closely related species for these novel taxa. This research contributes to fungal data from urban soils and enhances the understanding of the species diversity, taxonomy, and distribution of *Sympoventuriaceae*.

## Introduction

The family *Sympoventuriaceae* belongs to the order *Venturiales* in the class *Dothideomycetes* and was established by Zhang et al. (2011) based on a multigene phylogenetic analysis, morphological characteristics, and ecological habits. The type genus of the family is *Sympoventuria*, which was established by Crous et al. (2007) based on the type species *S. capensis*. Since the family was erected, the number of genera incorporated into it has continuously increased through ongoing morphological and molecular phylogenetic studies. Based on a comprehensive analysis of seven gene fragments (SSU, ITS, LSU, *act1*, *tub2*, *tef1*, and *rpb2*), Wei et al. (2022) confirmed that 22 genera belong to *Sympoventuriaceae*. Subsequently, Crous et al. (2024) established another new genus, *Zenophaeosphaeria*. To date, the family *Sympoventuriaceae* comprises 23 genera. Although the systematics of *Sympoventuriaceae* have become relatively stable, the delimitation of some genera and species still requires clarification through more newly collected specimens, multigene phylogenetic analyses, and phenotypic studies. For instance, the systematic positions of genera such as *Acroconidiellina* and *Matsushimaea* require further verification due to the lack of reliable molecular data or type strains. Members of *Sympoventuriaceae* exhibit high diversity in ecological habits, encompassing various lifestyles, such as saprobic, endophytic, plant pathogenic, and animal or human opportunistic pathogenic lifestyles (Satow et al. 2008; Seyedmousavi et al. 2013; Kidd et al. 2016; Zhang et al. 2018; Samerpitak et al. 2019; Benavent 2021; Murata et al. 2022). Ancestral trait reconstruction analysis suggested that the ancestral lifestyle of *Sympoventuriaceae* was saprobic and that parasitic lifestyles, such as plant pathogens and animal pathogens, evolved independently multiple times from saprobic ancestors (Wei et al. 2022).

The genus *Scolecobasidium* was erected by Abbott (1927) based on *S. terreum* (generic type) and *S. constrictum*. To date, *Scolecobasidium* is the largest genus in *Sympoventuriaceae*, comprising 56 species (Wei et al. 2022; Song et al. 2023; Zhang et al. 2024; Crous et al. 2026). This genus is characterized by brownish to black colonies, reduced, hyaline or pigmented conidiophores, and septate, smooth- or rough-walled, brown, single, dry, and rhexolytic conidia (Abbott 1927; Barron and Busch 1962; Seifert and Gams 2011). Members of *Scolecobasidium* are slow-growing and saprotrophic (Yasanthika et al. 2025), and they exhibit a worldwide distribution across diverse habitats, including soil, water, plants, humans, and fish or other cold-blooded vertebrates (Doty and Slater 1946; Ross and Yasutake 1973; Hamayun et al. 2009; Giraldo et al. 2014; Shen et al. 2020; Wei et al. 2022; Song et al. 2023; Zhang et al. 2024).

Samerpitak et al. (2014) transferred *Ochroconis gallopava* from *Ochroconis* to the newly established genus *Verruconis*, designating it as the type species. Subsequently, several new species have been introduced to the genus. Currently, 17 species are accepted in the genus, and all of them have sequence data available in GenBank (Wei et al. 2022; Shao et al. 2022; Bao et al. 2025). To date, the sexual morph has been reported only for *Verruconis mangrovei*, while all other species in this genus are described based on their asexual morphs (Bao et al. 2025). Members of *Verruconis* inhabit roots, leaves, tree latex, decaying wood, and soil (Zhang et al. 2018; Huanraluek et al. 2019; Qiao et al. 2019; Hernández-Restrepo et al. 2020; Shen et al. 2020; Shao et al. 2022; Wei et al. 2022; Yasanthika et al. 2025; Bao et al. 2025).

Arzanlou et al. (2007) removed *Veronaea simplex* from *Veronaea* and established *Veronaeopsis* with it as the type species. To date, this genus contains only a single species, *Veronaeopsis simplex*, with very few reliable strains and available molecular sequences (Wei et al. 2022). During an investigation of culturable fungi from urban soils in China, eight isolates representing *Sympoventuriaceae* were obtained. Morphological characteristics and multilocus (SSU, ITS, LSU, *tef1*, *tub2*, and *rpb2*) phylogeny were combined to determine their taxonomy. As a result, three novel species are proposed herein.

## Materials and мethods

### Fungal isolation and morphology

Soil samples were collected from green belts in three Chinese cities: (1) Guizhou Minzu University, Guiyang, Guizhou Province; (2) Guandu District, Kunming, Yunnan Province; and (3) Fuzhou Zoo, Fuzhou, Fujian Province. Samples were collected and stored in sterile Ziploc bags at 4 °C, transported to the laboratory, and processed immediately for experimentation. Fungi were isolated using the dilution method described by Tong et al. (2023, 2025).

Pure colonies on potato dextrose agar (PDA) medium were transferred to fresh PDA, oatmeal agar (OA), and synthetic nutrient-poor agar (SNA) plates and incubated at 25 °C in darkness for 14 days to assess colony morphology and macroscopic characteristics. The color of the colony was described using the color-coding system of the German Institute for Quality Assurance and Certification (Reichs-Ausschuss für Lieferbedingungen und Gütesicherung, RAL; https://www.ral-guetezeichen.de/), abbreviated as RAL in the text. The isolates were allowed to grow onto an adjacent, sterile coverslip that had been partially inserted into the agar surface at a 60° angle to observe fungal structures (Li et al. 2011), and 25% lactic acid was used as a mounting medium for microscopy. Morphological features were observed and recorded using a Zeiss Axio Imager A2 microscope and a Zeiss AxioCam MRc color digital camera (Carl Zeiss Ltd., München, Germany).

The ex-type living cultures of the novel taxa were preserved in the China General Microbiological Culture Collection Center (CGMCC). All living cultures were preserved in sterile 30% glycerol at −80 °C and deposited at the College of Eco-Environmental Engineering, Guizhou Minzu University. Dried culture specimens, dried at 50 °C, were deposited at the same institution. The taxonomic descriptions of the new taxa were registered in MycoBank.

### DNA extraction, PCR amplification, and sequencing

Genomic DNA was extracted from fungal mycelium growing on PDA plates for 14 days, according to the manufacturer’s protocol of the BioTeke Fungal Genomic DNA Extraction Kit (DP2032, BioTeke, Beijing, China). Six gene markers (SSU, ITS, LSU, *tef1*, *tub2*, and *rpb2*) were amplified and sequenced using the primer pairs listed in Table 1. The 25 μL PCR reactions contained 2.0 μL DNA template, 1.0 μL each of forward and reverse primers, 8.5 μL ddH_2_O, and 12.5 μL 2× MasterMix (Sangon Biotech, China). All newly generated sequences were submitted to GenBank (www.ncbi.nlm.nih.gov) (Table 2).

**Table 1.** Gene regions and respective primers used in the study.

Gene region	Primer pairs (forward/reverse)	Annealing temperature (°C)	Reference
ITS	ITS5	51	White et al. 1990
	ITS4		White et al. 1990
LSU	LR0R	51	Vilgalys and Hester 1990
	LR5		Vilgalys and Hester 1990
SSU	NS1	51	White et al. 1990
	NS4		White et al. 1990
*tef1*	983F	58	Rehner and Buckley 2005
	2218R		Rehner and Buckley 2005
	728	58	Carbone and Kohn 1999
	986		Carbone and Kohn 1999
*tub2*	Bt2a	55	Glass and Donaldson 1995
	Bt2b		Glass and Donaldson 1995
*rpb2*	5F	54	Liu et al. 1999
	7CR		Liu et al. 1999

**Table 2.** Strains and sequences of *Sympoventuriaceae* included in this study.

Species	Strain	GenBank and CNCB accession numbers					
		SSU	ITS	LSU	*tef1*	*tub2*	*rpb2*
*Acroconidiellina arecae*	NFCCI 3696		KX306747	KX306776			
*Bellamyces quercus*	CBS 46217 T		MK810901	MK810788	MK888726		MK887796
*Clavatispora thailandiaca*	MFLUCC 100107	KF770457	MH065721	KF770458			
*Echinocatena sinensis*	CGMCC 3.20775 T		OL897006	OL897048	ON568898	ON569026	ON568948
*Echinocatena sinensis*	GZUIFR 21.901		OL897007	OL897049	ON568899	ON569027	ON568949
*Fuscohilum rhodensis*	CBS 121641 T		MK810909	MK810796	MK888733	MK926471	MK887802
*Fuscohilum siciliana*	CBS 105.85 T	KP798640	MK810910	MK810797	MK888734	MK926472	MN091924
*Guizhoumyces aciculaea*	GUCC 18195 T	MZ503650	MZ503724	MZ503757	MZ546870	MZ546903	MZ546866
*Guizhoumyces aciculaea*	GUCC 18152	MZ503649	MZ503723	MZ503756	MZ546869	MZ546902	MZ546865
*Helicopsis olivaceum*	CBS 728.83	AY856925	MH861681	MH873393			
*Matsushimaea fasciculata*	GUCC 18239	MZ503651	MZ503725	MZ503758	MZ546871	MZ546904	MZ546867
*Matsushimaea monilioides*	CBS 143867 T		LT883468	LT883469			
*Melnikomyces longisporum*	HUGP 18226 T	MT731289	MT731290	MT731291	MT739516	MT739515	
*Melnikomyces thailandicus*	CBS 145767 T		MN794374	MN794351			
*Mycosisymbrium cirrhosum*	MTCC 12435	KR259885	KR259883	KR259884			KR349124
*Mycosisymbrium cirrhosum*	GUCC 1837	MZ503648	MZ503722	MZ503755	MZ546868	MZ546901	MZ546864
*Neocoleroa cameroonensis*	CBS 129041 T		MK810902	MK810789	MK888727	MN078219	MK887797
*Neocoleroa metrosideri*	ICMP 21139 T		KU131678	KU131677			
*Neofusicladium eucalypti*	CBS 128216 T		MK810903	MK810790	MK888728	MK926468	MK887798
*Neofusicladium eucalypticola*	CBS 141301 T		MK810904	MK810791	MK888729		MK887799
*Parafusicladium amoenum*	CBS 254.95 T		MK810906	MK810793	MK888730	MK926469	
*Parafusicladium paraamoenum*	CBS 141322 T		MK810908	MK810795	MK888732		MK887801
*Pinaceicola cordae*	CBS 126959 T		MK810911	MK810798	MK888735	MK926473	
*Pinaceicola pini*	CBS 463.82 T		MK810915	MK810802	MK888739	MK926477	MK887804
*Pseudosigmoidea excentrica*	CBS 469.95 T	NG_065605	HQ667543	KF282669	KF155975	MK926478	
*Pseudosigmoidea ibarakiensis*	NBRC 107891 T	NG_078726	LC146758	LC146759			
*Scolecobasidium acanthi*	CGMCC 3.24352 T		OQ448957	OQ448949	OQ442215	OQ442218	OQ442212
*Scolecobasidium aegiceratis*	CGMCC 3.24353 T		OQ448958	OQ448950	OQ442216	OQ442219	OQ442213
*Scolecobasidium ailanthi*	MFLUCC 17-0923 T	MK347838	MK347730	MK347947		MK412883	
*Scolecobasidium anellii*	CBS 284.64 T	KF156070	FR832477	KF156138	KF155995	KF156184	KF282684
*Scolecobasidium anomalum*	CBS 131816 T	KF156065	HE575201	KF156137	KF155986	KF156194	HE575205
*Scolecobasidium aquaticum*	CBS 140316 T	KX668260	KX668258	KX668259			
*Scolecobasidium bacilliforme*	CBS 100442 T	KP798638	KP798632	KP798635	KT272070	KT272059	
*Scolecobasidium blechni*	CBS 146055 T		MN562134	MN567641	MN556826	MN556843	
*Scolecobasidium burgersense*	CBS 151408 T		PP565150	PP532844			
*Scolecobasidium camellicola*	GUCC 18242 T	MZ503654	MZ503728	MZ503761	MZ546874	MZ546907	
***Scolecobasidium campestre***	**CGMCC 3. 29458 = ZY 24.010 T**	**PX522227**	**PX522232**	**PX522237**	**PX586637**	**PX586642**	**PX586632**
***Scolecobasidium campestre***	**ZY 24.011 T**	**PX522228**	**PX522233**	**PX522238**	**PX586638**	**PX586643**	**PX586633**
*Scolecobasidium capsici*	CBS 142096 T		KY173427	KY173518			
*Scolecobasidium coiledmyces*	GUCC 18245 T	MZ503657	MZ503731	MZ503764	MZ546877	MZ546910	
*Scolecobasidium constrictum*	CBS 202.27 T	KF156072	MH854929	MH866423	KF156003	KF156161	
*Scolecobasidium cordanae*	CBS 475.80 T	KF156058	KF156022	KF156122	KF155981	KF156197	
*Scolecobasidium crassihumicola*	CBS 120700	KJ867431	KJ867429	KJ867430	KJ867428	KJ867433	
*Scolecobasidium dracaenae*	CBS 141323 T		KX228283	KX228334	KX228377		KX228370
*Scolecobasidium echinulatum*	GUCC 18247 T	MZ503659	MZ503733	MZ503766	MZ546879	MZ546912	
*Scolecobasidium ellipsoideum*	GUCC 18264	MZ503676	MZ503750	MZ503783	MZ546896	MZ546929	
* Scolecobasidium.endophyticum *	CBS 149762 T		MN365755	MN308484	PX047958		
*Scolecobasidium ferulica*	IRAN3232C T		MF186874	MH400207			
*Scolecobasidium gamsii*	CBS 239.78 T	KF156088	KF156019	KF156150	KF155982	KF156190	
*Scolecobasidium globale*	CBS 119644 T	KF961108	KF961086	KF961097	KF961075	KF961065	
*Scolecobasidium guangxiense*	SS23 T	MK929277	MK934570	MK956169			
*Scolecobasidium guizhouense*	CGMCC 3.25502 T	OR680870	OR680501	OR680568	OR858900	OR843206	OR842918
*Scolecobasidium helicteris*	NFCCI 4310 T		MK014833			MK321318	
*Scolecobasidium humicola*	CBS 116655 T	KF156068	HQ667521	KF156124	KF155984	KF156195	
*Scolecobasidium icarus*	CBS 536.69 T	KF156084	HQ667524	KF156132	KF156009	KF156174	KF282700
*Scolecobasidium inverellipsoidisporum*	CGMCC 3.25504 T	OR680875	OR680506	OR680573	OR858905	OR843211	OR842923
*Scolecobasidium lascauxense*	CBS 131815 T	KF156069	FR832474	KF156136	KF155994	KF156183	FR832481
*Scolecobasidium laurentii*	NFCCI 5982 T		PV029878	PV029879	PV023971	PV023970	
*Scolecobasidium leishanicola*	GUCC 18259	MZ503671	MZ503745	MZ503778	MZ546891	MZ546924	
*Scolecobasidium longiphorum*	CGMCC 3.25505	OR680880	OR680511	OR680578	OR858910	OR843216	OR842928
*Scolecobasidium macrozamiae*	CBS 102491	KF156092	KF156021	KF156152	KF155983	KF156191	
*Scolecobasidium millerae*	BRIP75007a T		OP903485	OP903501			
*Scolecobasidium minimum*	CBS 510.71 T	KF156087	HQ667522	KF156134	KF156007	KF156172	
*Scolecobasidium mirabilis*	CBS 413.51 T	KF156076	HQ667536	KF156140	KF156001	KF156164	
*Scolecobasidium musae*	CBS 729.95 T	KF156082	KF156029	KF156144	KF155999	KF156171	
*Scolecobasidium musicola*	CBS 144441 T		MH327824	MH327860	MH327887	MH327898	MH327876
*Scolecobasidium nancywakeae*	BRIP72739c T		OQ917081	OQ892171			
*Scolecobasidium obovoideum*	GUCC 18246 T	MZ503658	MZ503732	MZ503765	MZ546878	MZ546911	
*Scolecobasidium olivaceum*	CBS 137170 T	LM644548	LM644521	LM644564	KT272067	LM644605	
*Scolecobasidium pandanicola*	CBS 140660 T		KT950850	KT950864			
*Scolecobasidium parahumicola*	CGMCC 3.25503 T	OR680873	OR680504	OR680571	OR858903	OR843209	OR842921
*Scolecobasidium patriciamatherae*	BRIP 75792a T		OR608746	OR602940			
*Scolecobasidium phaeophorum*	CBS 206.96 T	KP798637	KP798631	KP798634	KT272098	KT272062	KF282692
*Scolecobasidium podocarpi*	CBS 143174 T		MG386032	MG386085		MG386162	
*Scolecobasidium podocarpicola*	CBS 146057 T		MN562138	MN567645			MN556811
*Scolecobasidium ramosum*	CBS 137173 T	LM644551	LM644524	LM644567	KT272069	MZ546928	
*Scolecobasidium robustum*	CBS 112.97 T	KP798639	KP798633	KP798636	KT272071	KT272060	
*Scolecobasidium sexuale*	CBS 135765 T	KF156089	KF156018	KF156118	KF155976	KF156189	
*Scolecobasidium terrestre*	CBS 211.53 T	NG_062994	NR_145365	NG_058014	KF156005	KF156187	KF282686
*Scolecobasidium terreum*	CBS 203.27 T		HQ667544			HQ877665	KF282698
*Scolecobasidium tshawytschae*	CBS 228.66	KF156064	KF156016	KF156128	KF155992	KF156179	
*Scolecobasidium tshawytschae*	GUCC 18251	MZ503663	MZ503737	MZ503770	MZ546883	MZ546916	
*Scolecobasidium tshawytschae*	GUCC 18252	MZ503664	MZ503738	MZ503771	MZ546884	MZ546917	
*Scolecobasidium tshawytschae*	GUCC 18253	MZ503665	MZ503739	MZ503772	MZ546885	MZ546918	
*Scolecobasidium tshawytschae*	GUCC 18254	MZ503666	MZ503740	MZ503773	MZ546886	MZ546919	
*Scolecobasidium tshawytschae*	CBS 100438 T	KF156062	HQ667562	KF156126	KF155990	KF156180	KF282697
*Scolecobasidium tshawytschae*	CBS 130.65	KF156061	MH858517	MH870151	KF155989	KF156178	
*Scolecobasidium tshawytschae*	ZY 22.031	OR680879	OR680510	OR680577	OR858909	OR843215	OR842927
*Scolecobasidium tshawytschae*	CBS 454.77	KT272094		KT272089	KF155991	KF156181	
*Scolecobasidium tshawytschae*	CBS 850.73	KF156063	KT272079	KT272090	KF155988	KF156177	
*Scolecobasidium tshawytschae*	NBRC 32268	EU107353	DQ307334	EU107310	DQ307356		DQ415439
*Scolecobasidium verrucarium*	GUCC 18240 T	MZ503652	MZ503726	MZ503759	MZ546872	MZ546905	
*Scolecobasidium verrucosum*	CBS 383.81 T	KF156067	KF156015	KF156129	KT272099	KF156185	
*Scolecobasidium zunyiense*	GUCC 18241 T	MZ503653	MZ503727	MZ503760	MZ546873	MZ546906	
*Sterila eucalypti*	CBS 144019 T		MK810918	MK810805	MK888742		MK887807
*Sterila eucalypti*	CPC 14942		MK810916	MK810803	MK888740		MK887805
*Sympoventuria capensis*	CBS 120136 T	KF156094	MK810921	MK810808	MK888745	MK926481	MK887810
*Sympoventuria capensis*	CPC 12839		MK810922	MK810809	MK888746	MK926482	MK887811
*Troposporella fumosa*	CBS 351.94		MK810924	MH874121			
*Troposporella monilipes*	MUCL 19867	AY856920	DQ351723	AY856871			
*Veronaeopsis simplex*	CBS 588.66 T	KF156095	EU041820	EU041877			MN091925
***Veronaeopsis sinensis***	**CGMCC 3.29838 = ZY 25.010 T**	**PZ315057**	**PZ315066**	**PZ315075**	**PZ341956**	**PZ341947**	**PZ341941**
***Veronaeopsis sinensis***	**ZY 25.011**	**PZ315058**	**PZ315067**	**PZ315076**	**PZ341957**	**PZ341948**	**PZ341942**
***Veronaeopsis sinensis***	**ZY 25.012**	**PZ315059**	**PZ315068**	**PZ315077**	**PZ341958**	**PZ341949**	**PZ341943**
*Verruconis calidifluminalis*	CBS 125818 T	KF156046	AB385698	KF156108	KF155959	KF156202	
*Verruconis calidifluminalis*	CBS 125817	KF156045	AB385699	KF156107	KF155958	KF156201	
*Verruconis cylindricalis*	GUCC 18299 T	MZ503680	MZ503754	MZ503787	MZ546900	MZ546933	
*Verruconis gallopava*	CBS 437.64 T	KF156053	HQ667553	KF156112	KF155968	KF156203	KF282689
*Verruconis guizhouensis*	CGMCC 3.20874 T	ON764294	ON184018	ON764283			
*Verruconis hainanensis*	YMF 1.04165 T	MK248267	MK244397	MK248269			
*Verruconis heveae*	MFLUCC 17-0372 T	OL780530	MH602349	MH602348			
*Verruconis hyalina*	KUNCC 23–14194 T		PV138744	PV138599			
*Verruconis mangrovei*	NFCCI-4390 T	MN241147	MN782361	MN241144		MN848140	MN231836
*Verruconis pakchongensis*	TBRC-BCC 51642 T	OQ121937	OQ121928	OQ121946		OQ116768	OQ116751
*Verruconis panacis*	CBS 142802 T	MF536879	MF536882	MF536880		MF536883	
*Verruconis phayaoensis*	MFLUCC 17-0347 T	OL780531	OL780498	OL782077			
*Verruconis pseudotricladiata*	YMF 1.04915 T	MK248268	MK244396	MK248270		MK253013	
*Verruconis soli*	MFLUCC 22-0082 T	OP581426	OP581411	OP581410			
*Verruconis terricola*	CBS 131795 T		MK810925	MK810811			KC337072
*Verruconis thailandica*	CBS 145768 T		MN794375	MN794352			
*Verruconis thailandica*	GUCC 18267	MZ503679	MZ503753	MZ503786		MZ546932	
*Verruconis tricladiata*	NBRC 30208	EU107354		EU107286			DQ415436
*Verruconis verruculosa*	CBS 119775 T	KF156055	KF156014	KF156106	KF155974	KF156193	
***Verruconis obovatus***	**CGMCC 3.29459 = ZY25.007 T**	**PX522224**	**PX522229**	**PX522234**	**PX586634**	**PX586639**	**PX586629**
***Verruconis obovatus***	**ZY 25.008**	**PX522225**	**PX522230**	**PX522235**	**PX586635**	**PX586640**	**PX586630**
***Verruconis obovatus***	**ZY 25.009**	**PX522226**	**PX522231**	**PX522236**	**PX586636**	**PX586641**	**PX586631**
*Yunnanomyces pandanicola*	MFLUCC 17-2260 T	MH388333	MH388369	MH376743			MH412736
*Yunnanomyces sexualis*	CGMCC 3.25507 T		OR680512	OR680579	OR858911	OR843217	OR842929
*Zenophaeosphaeria calamagrostidis*	CBS 150822 T		PP791434	PP791462	PP780621		PP780614
***Tyrannosorus lichenicola***	CBS 144018 T		MK810953	MK810838	MK888775	MK926509	MK887840
***Tyrannosorus pinicola***	CBS 124.88 T	DQ471025	MK810951	MK810836		MK926507	DQ470928

Notes: T: Ex-type; DNA sequences for the new isolates are shown in bold. CBS: Westerdijk Fungal Biodiversity Institute, Utrecht, the Netherlands; CGMCC: China General Microbiological Culture Collection Center, Beijing, China; GUCC: Culture Collection of the Department of Plant Pathology, Agriculture College, Guizhou University, China; ICMP: International Collection of Micro-organisms from Plants, Landcare Research, Private Bag 92170, Auckland, New Zealand; IRAN: Fungal Culture Collections of the Iranian Research Institute of Plant Protection; MFLU (CC): Mae Fah Luang University Culture Collection, Chiang Rai, Thailand; MUCL: Université catholique de Louvain, Louvain-la-Neuve, Belgium; MTCC: Institute of Microbial Technology, Chandigarh, India; NBRC: Biological Resource Center; NFCCI: National Fungal Culture Collection of India, Pune, India.

### Phylogenetic analyses

Lasergene software (version 6.0, DNASTAR) was used to edit ambiguous bases at both ends of the raw forward and reverse reads and to assemble them. Sequences were aligned with MAFFT v7.037 (Katoh and Standley 2013), and alignments were trimmed with MEGA v6.06 (Tamura et al. 2013). The most appropriate models of sequence evolution for Bayesian inference and maximum likelihood analyses were selected using ModelFinder (Kalyaanamoorthy et al. 2017) in PhyloSuite v1.2.3 (Xiang et al. 2023).

The following three analyses were employed in this study: (1) an analysis based on the ITS + LSU + *tef1* + *tub2* + *rpb2* dataset to confirm the placement of the three novel taxa within the family *Sympoventuriaceae*, as well as single-gene phylogenetic analyses for each of these loci to evaluate species placement; (2) an analysis based on the SSU + ITS + LSU + *tub2* + *tef1* dataset to confirm the placement of *Scolecobasidium campestre* in the genus *Scolecobasidium*; and (3) an analysis based on the SSU + ITS + LSU + *tub2* dataset to confirm the placement of *Verruconis obovatus* in the genus *Verruconis*.

The maximum likelihood (ML) analysis was run in IQ-TREE v1.6.11 (Nguyen et al. 2015) with 10,000 bootstrap tests, using the ultrafast algorithm (Minh et al. 2013). The Bayesian inference (BI) analysis was conducted in MrBayes v3.2 (Ronquist et al. 2012) and in PhyloSuite v1.2.3 (Xiang et al. 2023). The Markov chain Monte Carlo (MCMC) method was used to perform 5 × 10^6^ simulations with a sampling frequency of 10^3^ generations and a 25% burn-in. After the analysis was finished, Tracer v1.5 (Drummond and Rambaut 2007) was used to determine burn-in and confirm that both runs had converged. Multilocus phylogenetic trees were opened and checked using FigTree v1.4.2 (Rambaut 2012), and the final trees were edited in Microsoft PowerPoint by inserting statistical supports from ML and BI.

## Results

### Phylogenetic analyses

Analysis 1

**Figure 1.**
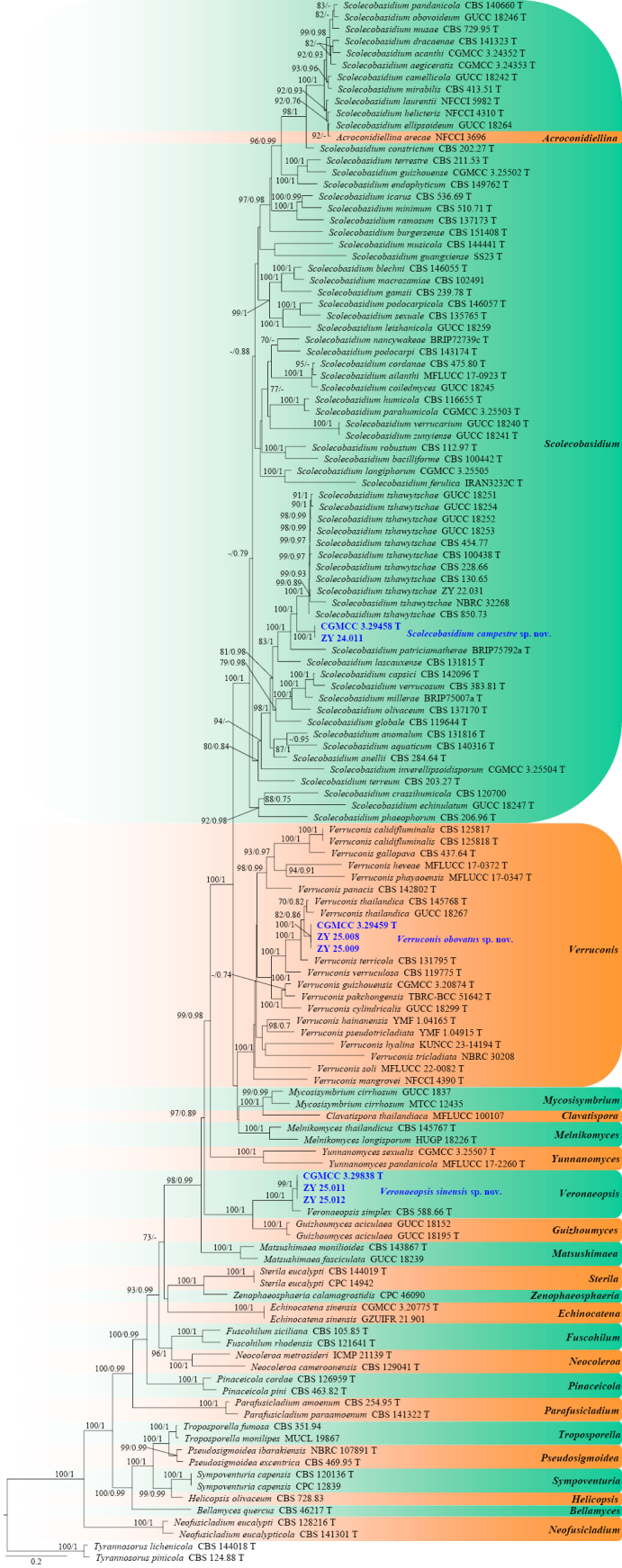
Phylogenetic tree inferred from a maximum likelihood analysis based on a concatenated alignment of ITS, LSU, *tef1*, *tub2*, and *rpb2* sequences from 131 isolates representing *Sympoventuriaceae* and outgroup taxa. Numbers at branches indicate support values (IQ-TREE-BS/BI-PP) above 70%/0.70. The new species are printed in blue. Strains with type status are indicated with “T.” The tree is rooted with *Tyrannosorus lichenicola* (CBS 144018) and *T. pinicola* (CBS 124.88).

The generic placements of the new isolates are confirmed in this analysis (Fig. 1). The sequence data comprised 131 taxa from *Sympoventuriaceae*, with *Tyrannosorus lichenicola* (CBS 144018) and *T. pinicola* (CBS 124.88) as the outgroup taxa. The dataset consisted of 2,959 characters (ITS, 482 bp; LSU, 822 bp; *tef1*, 378 bp; *tub2*, 478 bp; and *rpb2*, 799 bp). The best-fit model for the ML analysis was GTR+F+I+G4 for ITS, *tub2*, and *rpb2*; TN+F+I+G4 for LSU; and TIM2+F+I+G4 for *tef1*. The best-fit model for the BI analysis was GTR+F+I+G4 for ITS, LSU, *tef1*, *tub2*, and *rpb2*. In the phylogenetic tree, nearly all genera within *Sympoventuriaceae* formed monophyletic clades with high support, except for the genus *Acroconidiellina*, which was nested in the genus *Scolecobasidium* (Fig. 1). Additionally, isolates CGMCC 3.29458 and ZY 24.011 formed a stable clade in the genus *Scolecobasidium* and clustered sister to *S. patriciamatherae* and *S. tshawytschae* (Fig. 1). Isolates CGMCC 3.29459 and ZY 25.008–009 formed a stable clade in the genus *Verruconis* and clustered sister to *V. terricola*, *V. thailandica*, and *V. verruculosa* (Fig. 1). Isolates CGMCC 3.29838 and ZY 25.011–012 formed a stable clade in the genus *Veronaeopsis* and clustered sister to *V. simplex* (Fig. 1). Additionally, due to differences in dataset composition, although there were some discrepancies among the single-gene trees, as well as between the single-gene trees and the concatenated tree, the three proposed new species consistently formed independent lineages (Suppl. materials 1–5).

Analysis 2

Phylogenetic trees were generated in analysis 2 to establish the new species in the genus *Scolecobasidium* (Suppl. material 6). The sequence data comprised 70 taxa from *Scolecobasidium*, with *Fuscohilum siciliana* (CBS 105.85) and *Matsushimaea fasciculata* (GUCC 18239) as the outgroup taxa. The dataset consisted of 3,361 characters (SSU, 967 bp; ITS, 705 bp; LSU, 795 bp; *tub2*, 485 bp; and *tef1*, 409 bp). The best-fit model for the ML analysis was TIM2+F+I+G4 for SSU, ITS, LSU, and *tef1*; and TIM+I+G4 for *tub2*. The best-fit model for the BI analysis was GTR+F+I+G4 for SSU, ITS, LSU, and *tef1*; and GTR+I+G4 for *tub2*. In the phylogenetic tree, isolates CGMCC 3.29458 and ZY 24.011 formed a stable clade in the genus *Scolecobasidium* and clustered sister to *S. patriciamatherae* and *S. tshawytschae* (Suppl. material 6).

Analysis 3

Phylogenetic trees were generated in analysis 3 to establish the new species in the genus *Verruconis* (Suppl. material 7). The sequence data comprised 24 taxa from *Verruconis*, with *Scolecobasidium guizhouense* (CGMCC 3.25502) and *S. parahumicola* (CGMCC 3.25503) as the outgroup taxa. The dataset consisted of 2,735 characters (SSU, 962 bp; ITS, 541 bp; LSU, 778 bp; and *tub2*, 454 bp). The best-fit model for the ML analysis was HKY+I+G4 for SSU; GTR+F+I+G4 for ITS; TIM+F+I+G4 for LSU; and TPM3u+I+G4 for *tub2*. The best-fit model for the BI analysis was K2P+I+G4 for SSU; GTR+F+G4 for ITS; GTR+F+I+G4 for LSU; and K2P+I+G4 for *tub2*. In the phylogenetic tree, isolates CGMCC 3.29459 and ZY 25.008–009 formed a stable clade in the genus *Verruconis* and clustered sister to *V. terricola*, *V. thailandica*, and *V. verruculosa* (Suppl. material 7).

### Taxonomy

#### Scolecobasidium campestre

Taxon classificationFungiVenturialesSympoventuriaceae

H. Pan, Zhi Y. Zhang & G. Tao sp. nov.

6BE4B66D-B7E1-59C1-A33C-2B61974C4D30

MycoBank No: MB861297

Fig. 2

##### Etymology.

The epithet “*campestre*” (Latin) refers to the location where the type was collected, Guizhou Minzu University.

**Figure 2.**
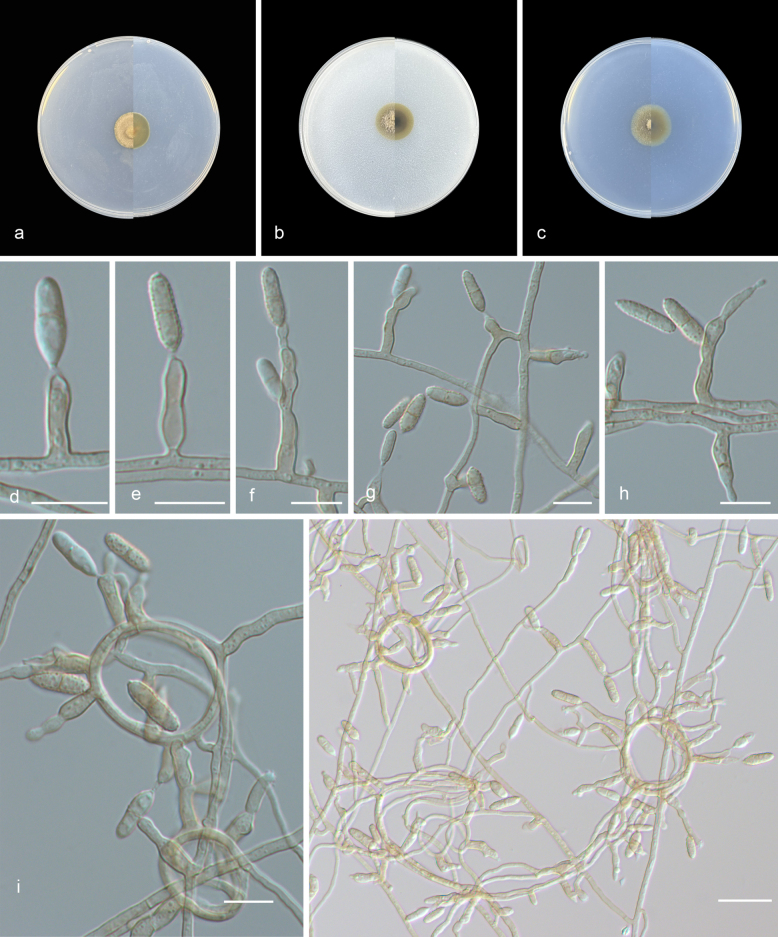
*Scolecobasidium campestre*. **a–c**. Upper and reverse views of cultures on PDA, OA, and SNA after 14 days at 25 °C; **d–h**. Conidiophores and conidiogenous cells bearing conidia; **i, j**. Conidiophores arising from hyphal coils and conidia. Scale bars: 10 μm (**d–i**), 20 μm (**j**).

##### Holotype.

China, Guizhou Province, Guiyang City, South Campus of Guizhou Minzu University, 26.37°N, 106.62°E, altitude: 1,174 m, soil, 23 July 2024, Heng Pan (holotype ZY H-24.010, dried culture; ex-type CGMCC 3.29458, *ibid*., ZY 24.010).

##### Description.

***Culture characteristics*** (14 d at 25 °C): Colony on PDA 18–19 mm diam., flat, felty, circular, aerial hyphae dense, slightly raised in the center, margin entire, green beige (RAL 1000); reverse olive yellow (RAL 1020). Colony on OA 19–20 mm diam., flat, felty, circular, aerial hyphae sparse at margin, margin entire, green beige (RAL 1000); reverse brown grey (RAL 7013) to green brown (RAL 8000). Colony on SNA, 20–22 mm diam., flat, hairy, slightly raised in the center, subcircular, margin entire, green beige (RAL 1000) to olive grey (RAL 7002); reverse brown grey (RAL 7013) to olive grey (RAL 7002).

***Hyphae*** septate, branched, hyaline to pale brown, smooth, and thin-walled, 1.5–3 μm wide, forming hyphal coils. Asexual morph: ***Conidiophores*** subcylindrical to cylindrical, arising from the aerial hyphae or hyphal coils, straight, aseptate, usually reduced to conidiogenous cells, (4.5–)6.5–22(–35) × 2–3.5 μm (av. ± SD = 12 ± 4.8 × 2.9 ± 0.2, *n* = 30). ***Conidiogenous cells*** solitary, occasionally branched, subhyaline to pale brown, clavate to subcylindrical, smooth, with single apical loci with rhexolytic conidiogenesis (2.5–)5.5–9 × 2–3.5 μm (av. ± SD = 6.6 ± 0.8 × 2.8 ± 0.2, *n* = 30). ***Conidia*** solitary, medianly 1-septate, subcylindrical, clavate or fusiform, smooth or rough with verruca, subhyaline to medium brown, 8.5–14.5 × 2.5–4 μm (av. ± SD = 11.6 ± 0.8 × 3.4 ± 0.2, *n* = 30). Sexual morph and chlamydospores were not observed.

##### Geographical distribution.

Guizhou Province, China.

##### Additional material examined.

China, Guizhou Province, Guiyang City, South Campus of Guizhou Minzu University, 26.37°N, 106.62°E, altitude: 1,174 m, soil, 23 July 2024, Heng Pan ZY 24.011.

##### Notes.

Phylogenetically, *Scolecobasidium campestre* is strongly supported (100/1) and clusters with *S. patriciamatherae* and *S. tshawytschae* (Fig. 1, Suppl. material 6). Morphologically, *S. campestre* differs from *S. tshawytschae* by having conidiophores that may arise from hyphal coils and are slightly larger (6.5–22 × 2–3.5 μm vs. 6.5–18.5 × 3–3.5 μm) (Samerpitak et al. 2014; Wei et al. 2022). The conidia of *S. campestre* are 1-septate and smaller (8.5–14.5 × 2.5–4 μm vs. 12.5–22 × 5–6 μm) (Samerpitak et al. 2014; Wei et al. 2022). *Scolecobasidium tshawytschae* produces acrogenous, ellipsoidal to fusoid, 0(–1)-septate chlamydospores, whereas chlamydospores are absent in *S. campestre* (Wei et al. 2022). Furthermore, due to the lack of morphological descriptive data for *S. patriciamatherae*, comparison with *S. campestre* was not possible (Tan and Shivas 2023a, 2023b, 2023c). However, *S. campestre* can also be distinguished from *S. patriciamatherae* and *S. tshawytschae* by their low sequence similarities (Table 3).

**Table 3.** Nucleotide comparisons of *Scolecobasidium campestre* (ex-type CGMCC 3.29458) with *S. patriciamatherae* (ex-type BRIP 75792a) and *S. tshawytschae* (ex-type CBS 100438).

Taxa	ITS	LSU	*tef1*	*tub2*	*rpb2*
*S. patriciamatherae* (BRIP 75792a)	216/723 bp (29.8%, 114 gaps)	29/869 bp (3.3%, 3 gaps)			
*S. tshawytschae* (CBS 100438)	221/760 bp (29%, 103 gaps)	20/801 bp (2.5%, 6 gaps)	71/433 bp (16.3%, 21 gaps)	67/485 bp (13.8%, no gap)	64/874 bp (7.3%, 3 gaps)

#### Verruconis obovatus

Taxon classificationFungiVenturialesSympoventuriaceae

M.Y. Zhang, Zhi Y. Zhang & G. Tao sp. nov.

B6409EBA-A97F-5EE0-BF9C-B79E6737B8E0

MycoBank No: MB861298

Fig. 3

##### Etymology.

The epithet “*obovatus*” (Latin) refers to the shape of conidia.

**Figure 3.**
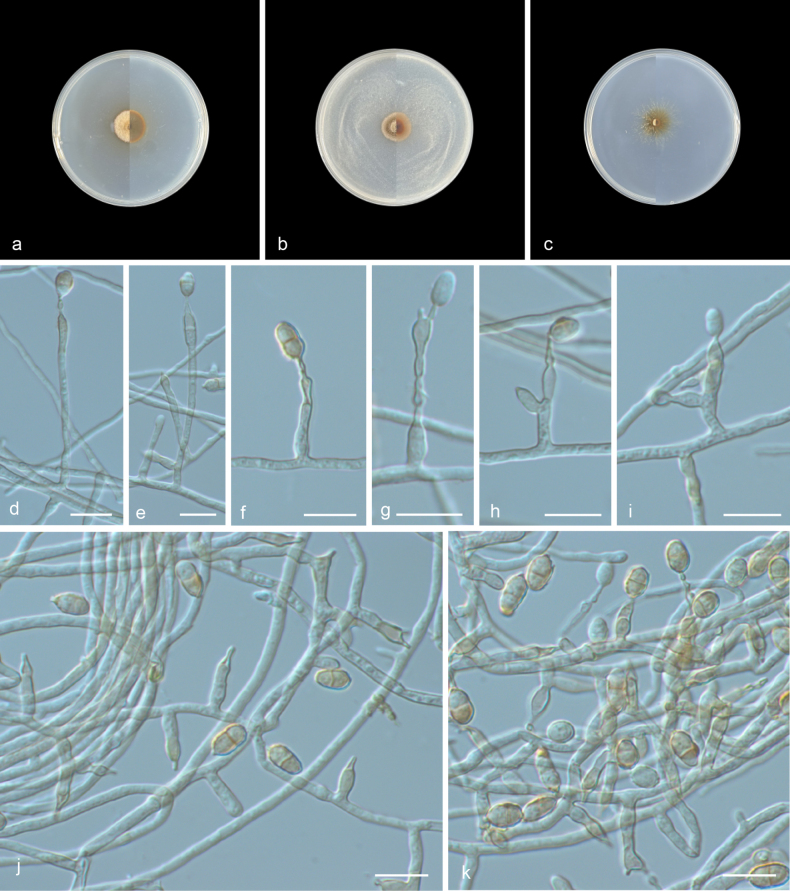
*Verruconis obovatus*. **a–c**. Upper and reverse views of cultures on PDA, OA, and SNA after 14 days at 25 °C; **d–k**. Conidiophores and conidiogenous cells bearing conidia. Scale bars: 10 μm (**d–k**).

##### Holotype.

China: Yunnan Province, Guandu District, 24.94°N, 102.77°E, altitude: 1,888 m, soil, 4 June 2024, Ming-Yi Zhang (holotype ZY H-25.007, dried culture; culture ex-type CGMCC 3.29459, *ibid*., ZY 25.007).

##### Description.

***Culture characteristics*** (14 d at 25 °C): Colony on PDA 18–19 mm diam., flat, cottony to velvety, aerial mycelium dense, margin entire, light ivory (RAL 1015); reverse ochre yellow (RAL 1024). Colony on OA 19–20 mm diam., flat to slightly raised, cottony to felty, subcircular, margin entire, beige (RAL 1001); reverse red brown (RAL 8012) to beige red (RAL 3012). Colony on SNA, 19–31 mm diam., flat to slightly raised at the center, aerial mycelium radial and sparse, brown pigment diffusing into the agar, margin fimbriate, light ivory (RAL 1015); reverse ochre yellow (RAL 1024).

***Hyphae*** septate, branched, hyaline to pale brown, smooth, and thin-walled, 1–2.5 μm wide. Asexual morph: ***Conidiophores*** erect, straight or slightly flexuous, aseptate, subcylindrical to cylindrical, usually reduced to conidiogenous cells, 3.5–24.5 (–38) × 2–3.5 μm (av. ± SD = 12.7 ± 4.5 × 2.7 ± 0.3, *n* = 30). ***Conidiogenous cells*** clavate or subcylindrical with apex slightly swollen, lateral, subhyaline, becoming pale brown toward apex, solitary, occasionally branched, smooth, thread-like apical loci with rhexolytic conidiogenesis 4–7.5(–13) × 2–3.5 μm (av. ± SD = 7 ± 1.8 × 2.7 ± 0.2, *n* = 30). ***Conidia*** medianly 1-septate, subhyaline to pale brown, smooth, ellipsoidal to subglobose or subcylindrical, both ends rounded, base occasionally papillate, 4–10 × 3.5–4.5 μm (av. ± SD = 7.1 ± 1.9 × 3.9 ± 0.4, *n* = 30). Sexual morph and chlamydospores were not observed.

##### Geographical distribution.

Yunnan Province, China.

##### Notes.

Multilocus phylogenetic analyses indicate that *Verruconis obovatus* is strongly supported (100/1) and clusters with *V. terricola*, *V. thailandica*, and *V. verruculosa* (Fig. 1, Suppl. material 7). Morphologically, the conidia of *V. obovatus* are ovoidal and do not constrict at the septum, whereas those of *V. thailandica* and *V. terricola* are broadly ellipsoidal and constrict at the septum; the conidia of *V. verruculosa* are oblong and constrict at the septum (Roy et al. 1962; Ren et al. 2013; Hernández-Restrepo et al. 2020). Additionally, *V. obovatus* can also be distinguished from *V. terricola*, *V. thailandica*, and *V. verruculosa* by their low sequence similarities (Table 4).

**Table 4.** Nucleotide comparisons of *Verruconis obovatus* (ex-type CGMCC 3.29459) with *V. terricola* (ex-type CBS 131795), *V. thailandica* (ex-type CBS 145768), and *V. verruculosa* (ex-type CBS 119775).

Taxa	ITS	LSU	*tef1*	*tub2*	*rpb2*
*V. terricola* (CBS 131795)	62/550 bp (11.3%, 27 gaps)	17/789 bp (2.2%, 4 gaps)			51/1,022 bp (4.9%, no gap)
*V. thailandica* (CBS 145768)	45/636 bp (7.1%, 20 gaps)	15/775 bp (1.9%, 1 gap)			
*V. verruculosa* (CBS 119775)	119/606 bp (19.6%, 40 gaps)	38/791 bp (4.8%, no gap)	176/469 bp (37.5%, 74 gaps)	55/471 bp (11.7%, 4 gaps)	

#### Veronaeopsis sinensis

Taxon classificationFungiVenturialesSympoventuriaceae

M.Y. Zhang, Zhi Y. Zhang & G. Tao sp. nov.

C9A8E542-8662-58E9-BDDE-4240AAC58E84

MycoBank No: MB863845

Fig. 4

##### Etymology.

The epithet “*sinensis*” (Latin) refers to the location where the type was collected, China.

**Figure 4.**
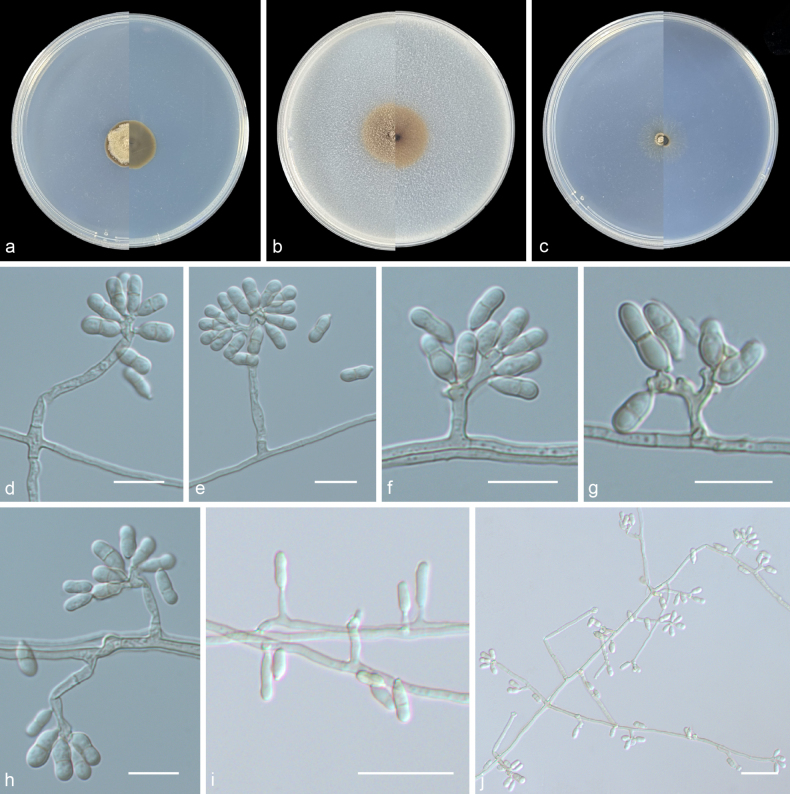
*Veronaeopsis sinensis*. **a–c**. Upper and reverse views of cultures on PDA, OA, and SNA after 14 days at 25 °C; **d**. Conidiogenous cells and conidia; **e–h**. Sympodially proliferating conidiogenous cells, rachis with crowded, prominent denticles, and conidia; **i**. Intercalary conidiogenous cells; **j**. Conidiophores, conidiogenous cells, and conidia. Scale bars: 10 μm (**d–h**); 20 μm (**i–j**).

##### Holotype.

China: Fujian Province, Fuzhou Zoo, 26.13°N, 119.28°E, altitude: 73 m, soil, 7 October 2025, Ming-Yi Zhang (holotype ZY H-25.010, dried culture; culture ex-type CGMCC 3.29838, *ibid*., ZY 25.010).

**Description. *Culture characteristics*** (14 d at 25 °C): Colony on PDA 20–21 mm diam., flat, dense, velvety, floccose, margin crenate, light ivory (RAL 1015); reverse curry (RAL 1027). Colony on OA 24–25 mm diam., flat, felty, circular, margin entire, beige (RAL 1001); reverse beige (RAL 1001). Colony on SNA, 19–20 mm diam., flat, hairy, slightly raised in the center, subcircular, aerial hyphae sparse at margin, green beige (RAL 1000); reverse moss grey (RAL 7003) to green beige (RAL 1000).

***Hyphae*** septate, branched, smooth, hyaline to subhyaline, 1.5–3 μm wide. Asexual morph: ***Conidiophores*** arising vertically or at an acute angle from aerial hyphae, lateral or intercalary, simple, occasionally branched, 0–1-septate, often reduced to conidiogenous cells, hyaline to subhyaline, 12–31.5 × 1–2.5 μm (av. ± SD = 18.1 ± 3 × 1.9 ± 0.2, *n* = 30). ***Conidiogenous cells*** arising from aerial hyphae, lateral, intercalary, or terminal, occasionally terminally integrated in the conidiophores, sometimes with a single apical denticle, smooth, hyaline to subhyaline, 1.5–29 × 0.5–2.5 μm (av. ± SD = 12.2 ± 2.3 × 1.5 ± 0.2, *n* = 30), rachis generally straight or irregularly geniculate, with crowded, prominent denticles. ***Conidia*** solitary, hyaline to subhyaline, smooth, ellipsoid, obovoid, oblong-ellipsoidal to subcylindrical, (0–)1-septate, slightly constricted at the septum, 3.5–11.5 × 2.5–3.5 μm (av. ± SD = 7.8 ± 0.8 × 2.9 ± 0.2, *n* = 30). Sexual morph: not observed.

##### Geographical distribution.

Fujian Province, China.

##### Additional material examined.

China, Fujian Province, Fuzhou City, Fuzhou Zoo, 26.14°N, 119.28°E, altitude: 107 m, soil, 7 October 2025, Ming-Yi Zhang ZY 25.011, *ibid*. ZY 25.012.

##### Notes.

Multilocus phylogenetic analyses indicate that *Veronaeopsis sinensis* is strongly supported (99/1) and clusters with *V. simplex* (Fig. 1). Morphologically, *V. sinensis* is similar to *V. simplex*, but the conidiophores of *V. sinensis* are simple, rarely branched, 0–1-septate, and its conidia are 3.5–11.5 × 2.5–3.5 μm (av. 7.8 × 2.9 μm); whereas in *V. simplex*, the conidiophores are simple or branched, and the conidia measure (6–)10–12(–15) × (2–)2.5–3(–4) μm (Arzanlou et al. 2007). Additionally, they can be distinguished by their low sequence similarities. Based on a pairwise comparison of ITS, LSU, and *rpb2*, *V. sinensis* (ex-type CGMCC 3.29838) differs from *V. simplex* (ex-type CBS 588.66) by 4.7% (22/461 bp, six gaps) in ITS, 2% (17/825 bp, nine gaps) in LSU, and 3.4% (28/804 bp, no gaps) in *rpb2*.

## Discussion

As primary decomposers, fungi drive the mineralization and humification of organic matter, thereby sustaining soil fertility in urban green spaces, such as parks, gardens, and roadside trees (Newbound et al. 2010; Zhang et al. 2019). Research on urban soil fungi has mainly focused on the following aspects: exploring the impact of urbanization gradients on the diversity and composition of fungal communities (Abrego et al. 2020; Rusterholz and Baur 2023); analyzing differences in fungal communities across various types of urban green spaces (e.g., parks, lawns, roadside tree pits, and agricultural fields) (McGuire et al. 2013; Gómez-Brandón et al. 2022); and investigating how urban environmental factors, such as soil physicochemical properties, pollutants, and management practices, shape specific functional groups, such as mycorrhizal and pathogenic fungi (Salomon et al. 2020; Scholier et al. 2023). However, the taxonomic study of urban soil fungi has not received sufficient attention. Recently, during a project on the diversity of keratinophilic fungi in urban soils of China, the isolated taxa were taxonomically characterized (Zhang et al. 2021, 2023, 2024; Pan et al. 2025; Wang et al. 2025). Similarly, Crous et al. (2021) surveyed the culturable fungi in garden soils of the Netherlands through a citizen science project. These few studies have revealed a substantial number of undescribed fungal taxa residing in urban environments. In this study, combining morphological characteristics with multigene phylogenetic analyses, three new species of *Sympoventuriaceae* (*Scolecobasidium campestre*, *Verruconis obovatus*, and *Veronaeopsis sinensis*) are reported. This contributes to fungal data from urban soils and enhances the understanding of the species diversity, taxonomy, and distribution of *Sympoventuriaceae*.

Urbanization has swept across the globe over the last several decades, inevi­tably resulting in increased anthropogenic changes to the environment (Berry 2008). These human activities, construction projects, and distinct land management practices profoundly impact urban soil fungi. However, although urban soils are heavily disturbed and managed environments, their topography and complex human-induced alterations shape exceptionally high heterogeneity. Such urban soils may provide highly, heterogeneous niches that support active microbial communities, potentially fostering unique and undescribed fungi that diverge significantly from those in natural habitats. Therefore, urban environments act as overlooked reservoirs of fungal diversity. Nevertheless, whether these newly discovered taxa are uniquely adapted to urban environments or are simply ubiquitous soil generalists remains an open question. Fungi play essential roles in ecosystem processes and functions, yet research on soil fungi, particularly concerning urban environments, remains in its infancy (Hyde et al. 2024). Because the current literature regarding comprehensive investigations of fungal taxa in urban soil is still limited, the successful isolation of these specific new taxa at this stage carries a degree of sampling serendipity. It is currently difficult to definitively conclude their exact ecological adaptations due to the limited sampling scale. Future systematic and in-depth investigations across broader urban soil habitats are required to verify whether these novel taxa exhibit specialized urban adaptation or represent incidental isolates from broadly distributed soil communities.

To date, most of the 23 genera in the family *Sympoventuriaceae* are relatively small, consisting of only a few species or even a single species, such as *Bellamyces*, *Clavatispora*, *Guizhoumyces*, *Mycosisymbrium*, *Sterila*, *Veronaeopsis*, and *Zenophaeosphaeria* (Wei et al. 2022). Furthermore, reliable strains and credible molecular data are scarce for some genera, so the current classification system may not accurately reflect reality. For example, in the phylogenetic tree (Fig. 1), *Acroconidiellina* is nested within *Scolecobasidium*. Currently, *Acroconidiellina* comprises four species (i.e., *A. arecae*, *A. chloridis*, *A. loudetiae*, and *A. urtiagae*), but DNA molecular data are available only for *A. arecae*. Therefore, the primary reason for this phenomenon may be that *A. arecae* is currently represented solely by ITS and LSU sequences, and these two genes exhibit low resolution at the generic level. Among them, only ITS provides some nucleotide differences, which do not reflect the true intergeneric relationships. In the future, collecting more specimens, isolating more species and strains, and obtaining more molecular sequences will help establish a modern taxonomic system for this family that is closer to nature. The genus *Veronaeopsis* previously comprised only one species, *V. simplex*. This study reports another species of the genus, *V. sinensis*, further solidifying the taxonomic status of the genus.

Currently, fungal taxonomy stands at a crossroads regarding whether detailed morphological descriptions are mandatory or whether reliance on molecular sequences alone suffices. Some studies have established new taxa based solely on molecular sequence differences and phylogenetic analyses, such as *Scolecobasidium millerae*, *S. nancywakeae*, and *S. patriciamatherae* (Tan and Shivas 2022, 2023a, b). Until consensus is reached, the establishment of new species within groups that produce spores in natural environments—or even under cultivation conditions—should include morphological descriptions to avoid confusion arising from differing interpretations, such as *Bisifusarium sinense* (Tan and Shivas 2023c; Zhang et al. 2024, 2025). According to the new species delineation criteria proposed by Jeewon and Hyde (2016), phylogenetic analyses should include the ITS region and at least one protein-coding gene. Unfortunately, the establishment of *S. millerae*, *S. nancywakeae*, and *S. patriciamatherae* does not meet these criteria. Furthermore, if new species-level taxa are to be established based on molecular sequence differences and phylogenetic analyses, the phylogenetic analysis should at least be conducted at a higher taxonomic level (genus level), meaning that molecular data from as many genera within the family as possible should be included to avoid erroneous results. For example, Tan and Shivas (2024) established *Chlamydocillium margaretcollinsiae* based on sequence differences and phylogenetic analyses with limited molecular data sampling. Recently, Zhao et al. (2026), using large-scale molecular data sampling within *Hypocreales*, demonstrated that *C. margaretcollinsiae* belongs to *Polyphialocladium* rather than *Chlamydocillium*. The molecular data used in this study cover 23 genera of *Sympoventuriaceae*, supporting the robustness of the results.

## Supplementary Material

XML Treatment for Scolecobasidium campestre

XML Treatment for Verruconis obovatus

XML Treatment for Veronaeopsis sinensis

## Additional information

### Artificial Intelligence (AI) use

The authors accept full responsibility for the content of the manuscript, including the disclosure of any use of AI.

No AI tools were used in the preparation of this manuscript.

### Author ORCIDs

M.-Y. Zhang https://orcid.org/0009-0000-0691-3941

H. Pan https://orcid.org/0009-0003-7472-3199

Y.-L. Ren https://orcid.org/0000-0003-2424-1266

Y.-J. Wu https://orcid.org/0009-0005-1316-5546

Z.-Q. Fang https://orcid.org/0009-0001-0963-1760

B.-D. Sun https://orcid.org/0000-0003-0272-6422

G.Tao https://orcid.org/0000-0002-0882-3752

Z.-Y. Zhang https://orcid.org/0000-0003-2031-7518

### Data availability

All of the data that support the findings of this study are available in the main text or Supplementary Information.

## Supplementary materials

Supplementary material 1 Phylogenetic tree inferred from a maximum likelihood analysis based on ITS sequences from 129 isolates representing *Sympoventuriaceae* and outgroup taxa Data type pdf Explanation note Numbers at branches indicate support values (IQ-TREE-BS/BI-PP) above 70%/0.70. The new species are printed in blue. Strains with type status are indicated with “T.” The tree is rooted with *Tyrannosorus lichenicola* (CBS 144018) and *T. pinicola* (CBS 124.88). https://binary.pensoft.net/file/1669863This dataset is made available under the Open Database License (http://opendatacommons.org/licenses/odbl/1.0/). The Open Database License (ODbL) is a license agreement intended to allow users to freely share, modify, and use this Dataset while maintaining this same freedom for others, provided that the original source and author(s) are credited. Ming-Yi Zhang, Heng Pan, Yu-Lian Ren, Yun-Jie Wu, Zhi-Qing Fang, Bing‑Da Sun, Gang Tao, Zhi-Yuan Zhang

Supplementary material 2 Phylogenetic tree inferred from a maximum likelihood analysis based on LSU sequences from 129 isolates representing *Sympoventuriaceae* and outgroup taxa Data type pdf Explanation note Numbers at branches indicate support values (IQ-TREE-BS/BI-PP) above 70%/0.70. The new species are printed in blue. Strains with type status are indicated with “T.” The tree is rooted with *Tyrannosorus lichenicola* (CBS 144018) and *T. pinicola* (CBS 124.88). https://binary.pensoft.net/file/1669864This dataset is made available under the Open Database License (http://opendatacommons.org/licenses/odbl/1.0/). The Open Database License (ODbL) is a license agreement intended to allow users to freely share, modify, and use this Dataset while maintaining this same freedom for others, provided that the original source and author(s) are credited. Ming-Yi Zhang, Heng Pan, Yu-Lian Ren, Yun-Jie Wu, Zhi-Qing Fang, Bing‑Da Sun, Gang Tao, Zhi-Yuan Zhang

Supplementary material 3 Phylogenetic tree inferred from a maximum likelihood analysis based on *tef1* sequences from 87 isolates representing *Sympoventuriaceae* and outgroup taxa Data type pdf Explanation note Numbers at branches indicate support values (IQ-TREE-BS/BI-PP) above 70%/0.70. The new species are printed in blue. Strains with type status are indicated with “T.” The tree is rooted with *Tyrannosorus lichenicola* (CBS 144018). https://binary.pensoft.net/file/1669865This dataset is made available under the Open Database License (http://opendatacommons.org/licenses/odbl/1.0/). The Open Database License (ODbL) is a license agreement intended to allow users to freely share, modify, and use this Dataset while maintaining this same freedom for others, provided that the original source and author(s) are credited. Ming-Yi Zhang, Heng Pan, Yu-Lian Ren, Yun-Jie Wu, Zhi-Qing Fang, Bing‑Da Sun, Gang Tao, Zhi-Yuan Zhang

Supplementary material 4 Phylogenetic tree inferred from a maximum likelihood analysis based on *tub2* sequences from 91 isolates representing *Sympoventuriaceae* and outgroup taxa Data type pdf Explanation note Numbers at branches indicate support values (IQ-TREE-BS/BI-PP) above 70%/0.70. The new species are printed in blue. Strains with type status are indicated with “T.” The tree is rooted with *Tyrannosorus lichenicola* (CBS 144018) and *T. pinicola* (CBS 124.88). https://binary.pensoft.net/file/1669866This dataset is made available under the Open Database License (http://opendatacommons.org/licenses/odbl/1.0/). The Open Database License (ODbL) is a license agreement intended to allow users to freely share, modify, and use this Dataset while maintaining this same freedom for others, provided that the original source and author(s) are credited. Ming-Yi Zhang, Heng Pan, Yu-Lian Ren, Yun-Jie Wu, Zhi-Qing Fang, Bing‑Da Sun, Gang Tao, Zhi-Yuan Zhang

Supplementary material 5 Phylogenetic tree inferred from a maximum likelihood analysis based on *rpb2* sequences from 57 isolates representing *Sympoventuriaceae* and outgroup taxa Data type pdf Explanation note Numbers at branches indicate support values (IQ-TREE-BS/BI-PP) above 70%/0.70. The new species are printed in blue. Strains with type status are indicated with “T.” The tree is rooted with *Tyrannosorus lichenicola* (CBS 144018) and *T. pinicola* (CBS 124.88). https://binary.pensoft.net/file/1669867This dataset is made available under the Open Database License (http://opendatacommons.org/licenses/odbl/1.0/). The Open Database License (ODbL) is a license agreement intended to allow users to freely share, modify, and use this Dataset while maintaining this same freedom for others, provided that the original source and author(s) are credited. Ming-Yi Zhang, Heng Pan, Yu-Lian Ren, Yun-Jie Wu, Zhi-Qing Fang, Bing‑Da Sun, Gang Tao, Zhi-Yuan Zhang

Supplementary material 6 Phylogenetic tree inferred from a maximum likelihood analysis based on a concatenated alignment of SSU, ITS, LSU, *tub2*, and *tef1* sequences from 70 isolates representing the genus *Scolecobasidium* and outgroup taxa Data type pdf Explanation note Numbers at branches indicate support values (IQ-TREE-BS/BI-PP) above 70%/0.70. The new species are printed in blue. Strains with type status are indicated with “T.” The tree is rooted with *Fuscohilum siciliana* (CBS 105.85) and *Matsushimaea fasciculata* (GUCC 18239). https://binary.pensoft.net/file/1669868This dataset is made available under the Open Database License (http://opendatacommons.org/licenses/odbl/1.0/). The Open Database License (ODbL) is a license agreement intended to allow users to freely share, modify, and use this Dataset while maintaining this same freedom for others, provided that the original source and author(s) are credited. Ming-Yi Zhang, Heng Pan, Yu-Lian Ren, Yun-Jie Wu, Zhi-Qing Fang, Bing‑Da Sun, Gang Tao, Zhi-Yuan Zhang

Supplementary material 7 Phylogenetic tree inferred from a maximum likelihood analysis based on a concatenated alignment of SSU, ITS, LSU, and *tub2* sequences from 24 isolates representing the genus *Verruconis* and outgroup taxa Data type pdf Explanation note Numbers at branches indicate support values (IQ-TREE-BS/BI-PP) above 70%/0.70. The new species are printed in blue. Strains with type status are indicated with “T.” The tree is rooted with *Scolecobasidium parahumicola* (CGMCC 3.25503) and *S. guizhouense* (CGMCC 3.25502). https://binary.pensoft.net/file/1669869This dataset is made available under the Open Database License (http://opendatacommons.org/licenses/odbl/1.0/). The Open Database License (ODbL) is a license agreement intended to allow users to freely share, modify, and use this Dataset while maintaining this same freedom for others, provided that the original source and author(s) are credited. Ming-Yi Zhang, Heng Pan, Yu-Lian Ren, Yun-Jie Wu, Zhi-Qing Fang, Bing‑Da Sun, Gang Tao, Zhi-Yuan Zhang
