# Supplementary figures and images for: New additions to Sympoventuriaceae (Venturiales, Dothideomycetes): three new species from China
Source: MycoKeys. 2026 Jun 5;133:209–35. doi: 10.3897/mycokeys.133.197363 (PMC13263719; doi:10.3897/mycokeys.133.197363)

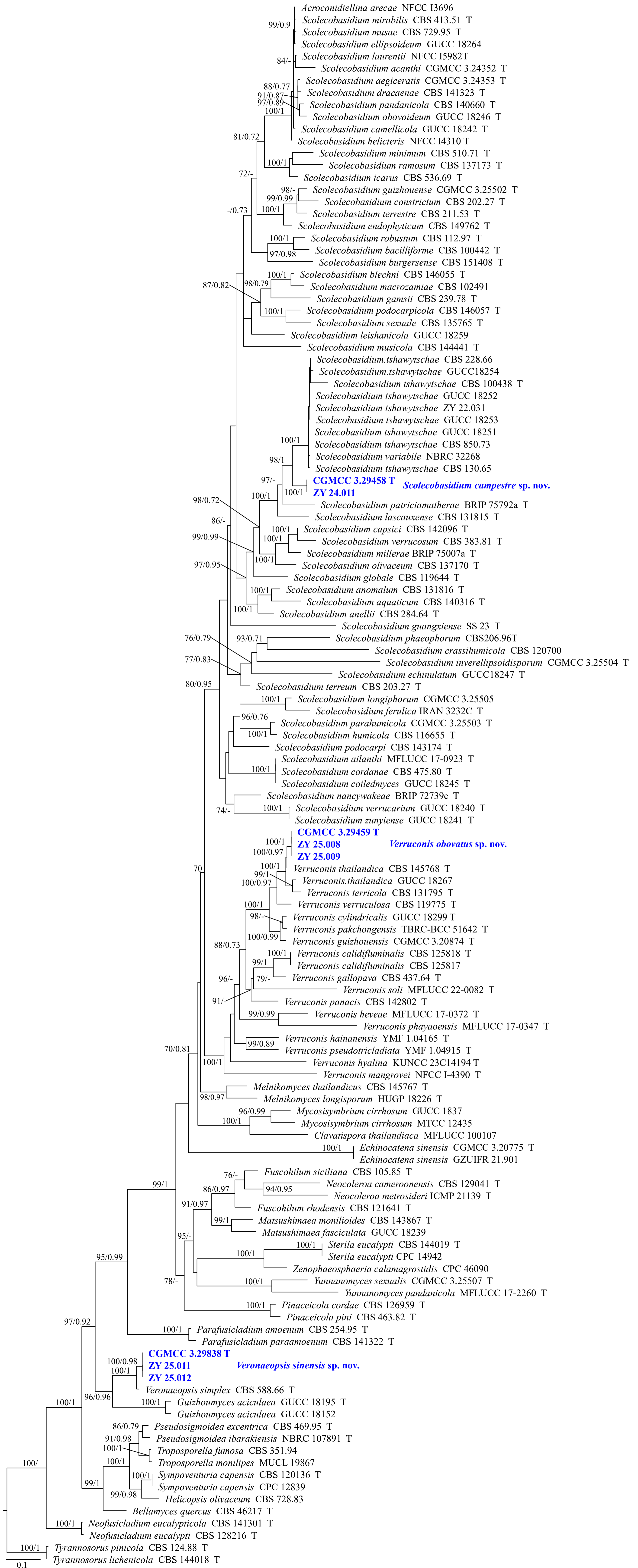

Supplement: Supplementary material 1 — Phylogenetic tree inferred from a maximum likelihood analysis based on ITS sequences from 129 isolates representing Sympoventuriaceae and outgroup taxa [file mycokeys-133-209-s001.pdf]

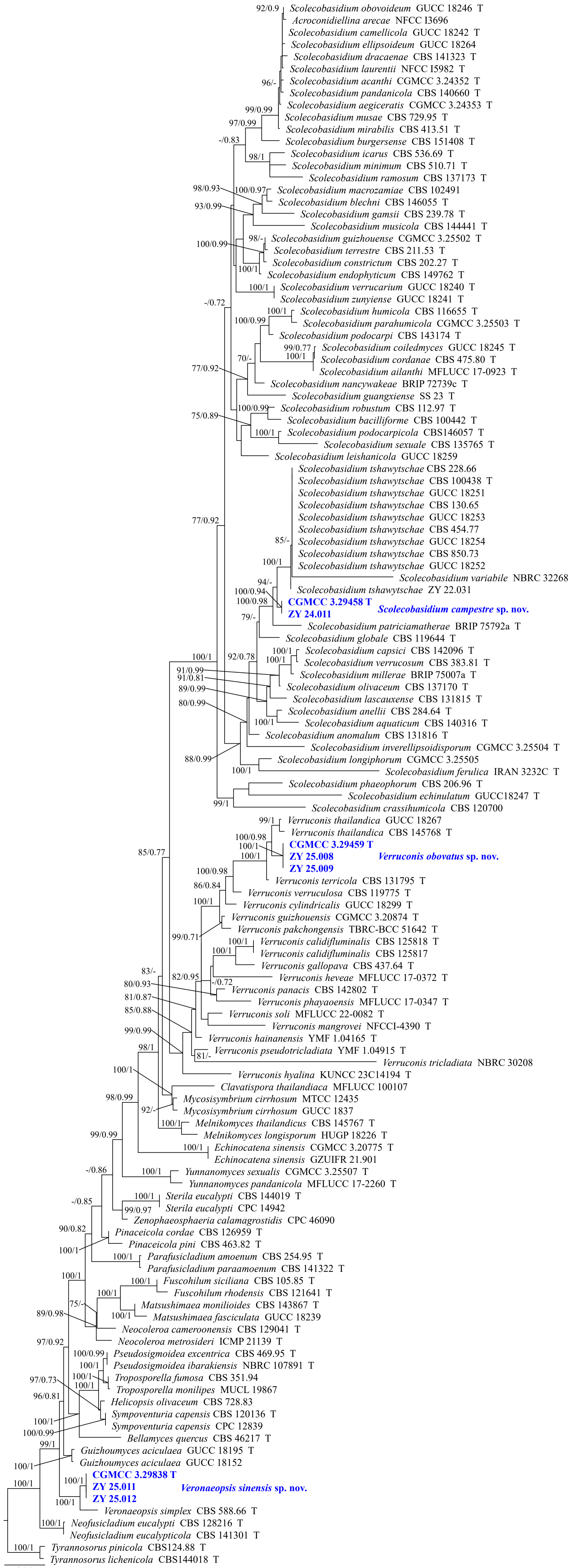

Supplement: Supplementary material 2 — Phylogenetic tree inferred from a maximum likelihood analysis based on LSU sequences from 129 isolates representing Sympoventuriaceae and outgroup taxa [file mycokeys-133-209-s002.pdf]

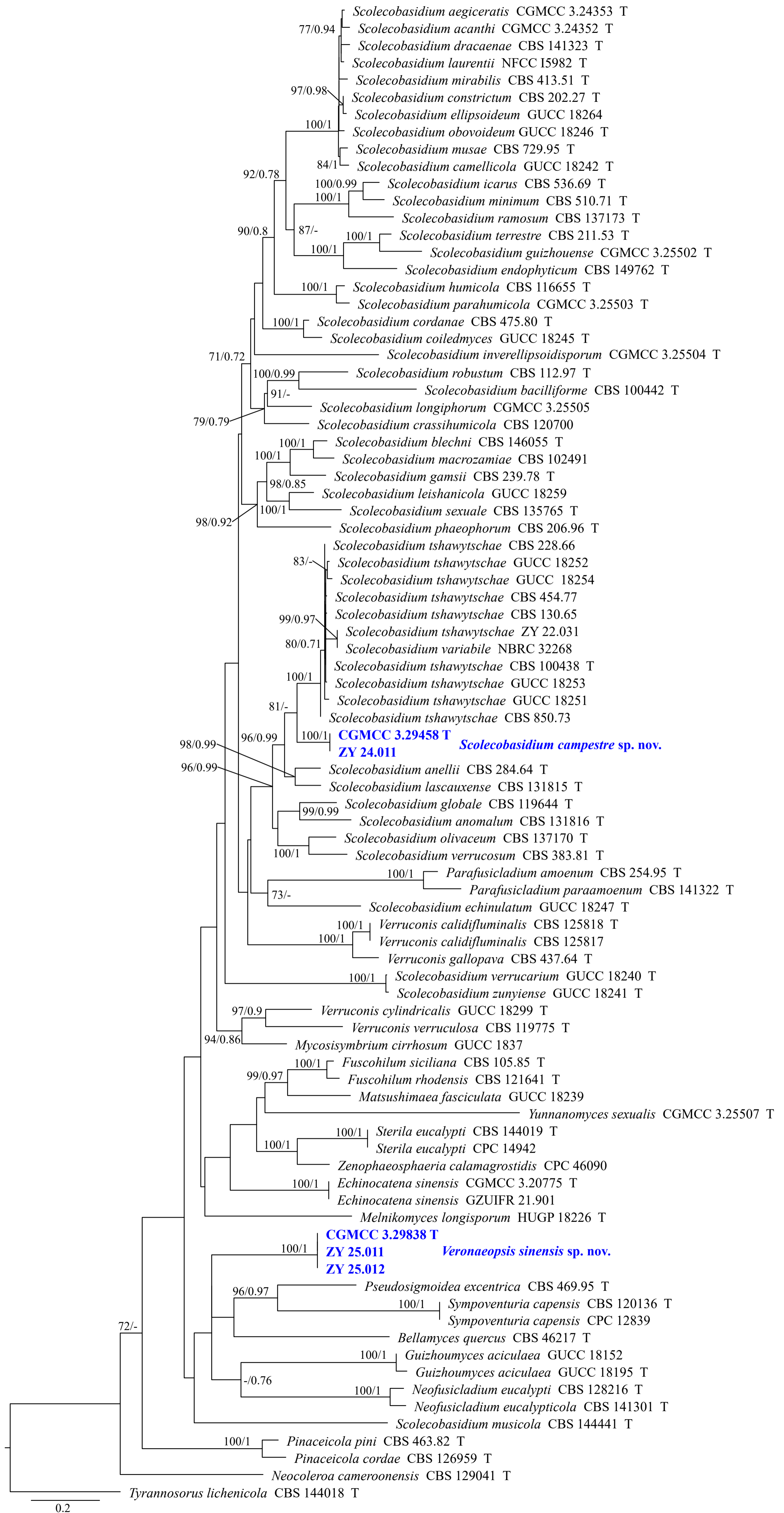

Supplement: Supplementary material 3 — Phylogenetic tree inferred from a maximum likelihood analysis based on tef1 sequences from 87 isolates representing Sympoventuriaceae and outgroup taxa [file mycokeys-133-209-s003.pdf]

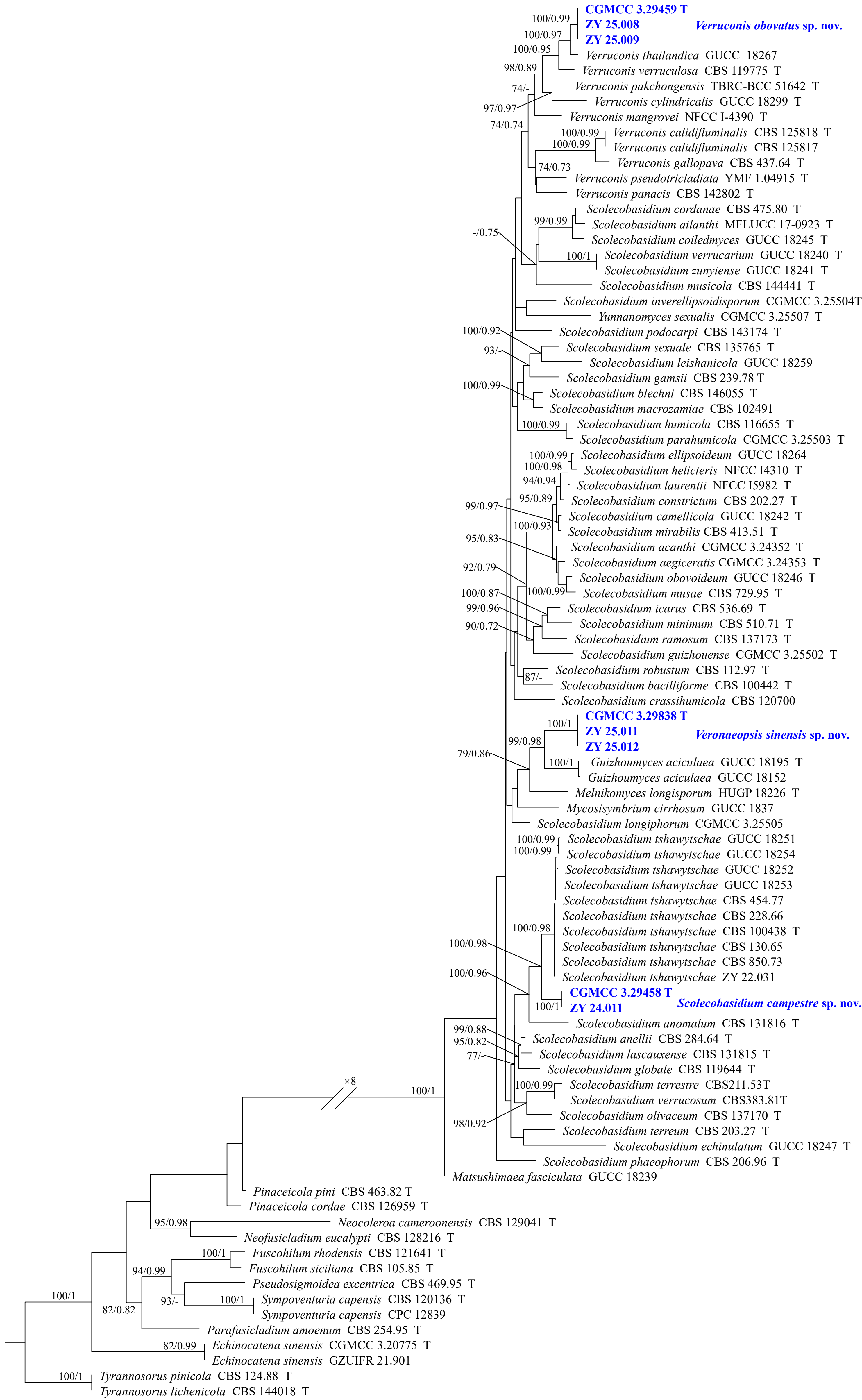

Supplement: Supplementary material 4 — Phylogenetic tree inferred from a maximum likelihood analysis based on tub2 sequences from 91 isolates representing Sympoventuriaceae and outgroup taxa [file mycokeys-133-209-s004.pdf]

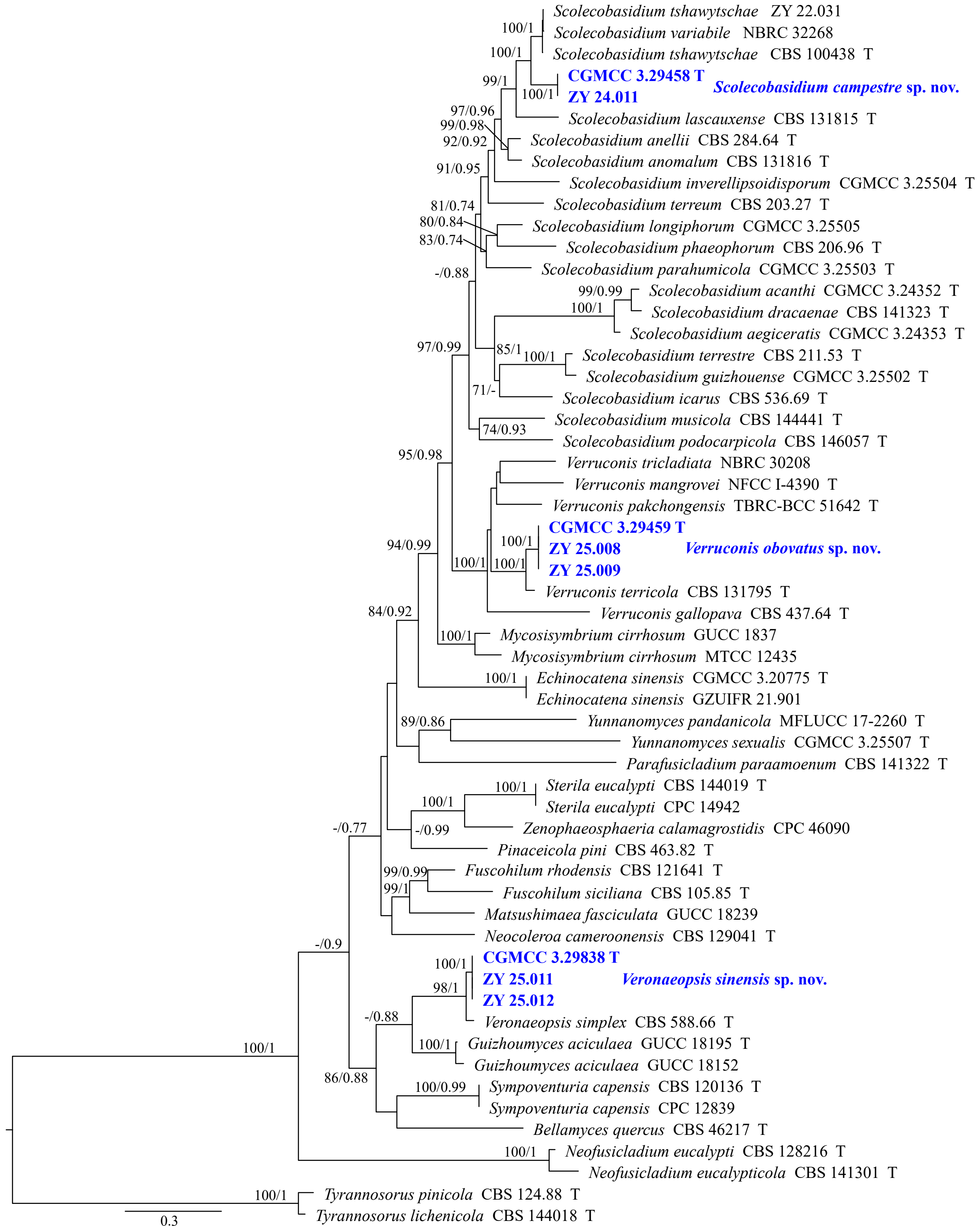

Supplement: Supplementary material 5 — Phylogenetic tree inferred from a maximum likelihood analysis based on rpb2 sequences from 57 isolates representing Sympoventuriaceae and outgroup taxa [file mycokeys-133-209-s005.pdf]

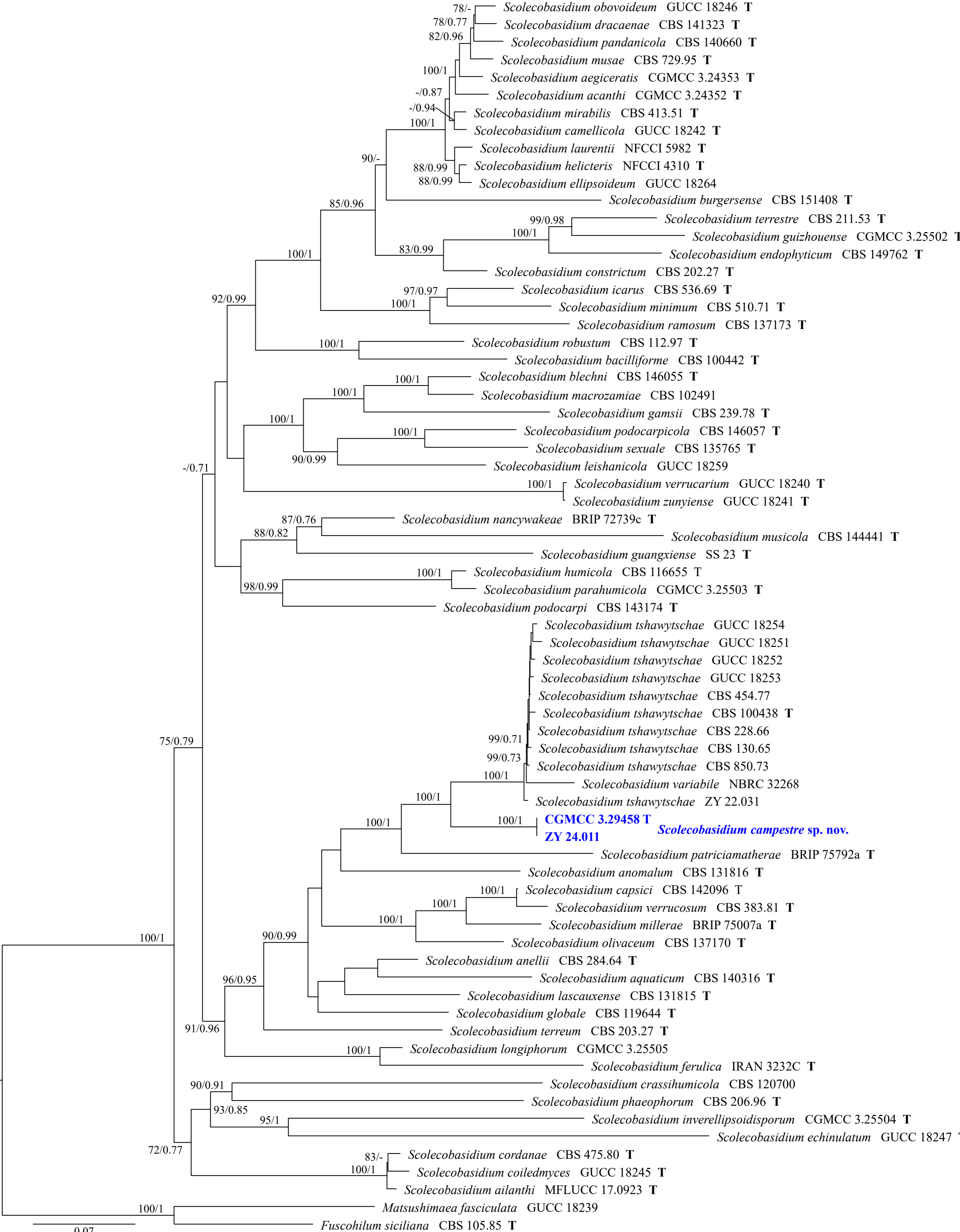

Supplement: Supplementary material 6 — Phylogenetic tree inferred from a maximum likelihood analysis based on a concatenated alignment of SSU, ITS, LSU, tub2, and tef1 sequences from 70 isolates representing the genus Scolecobasidium and outgroup taxa [file mycokeys-133-209-s006.pdf]

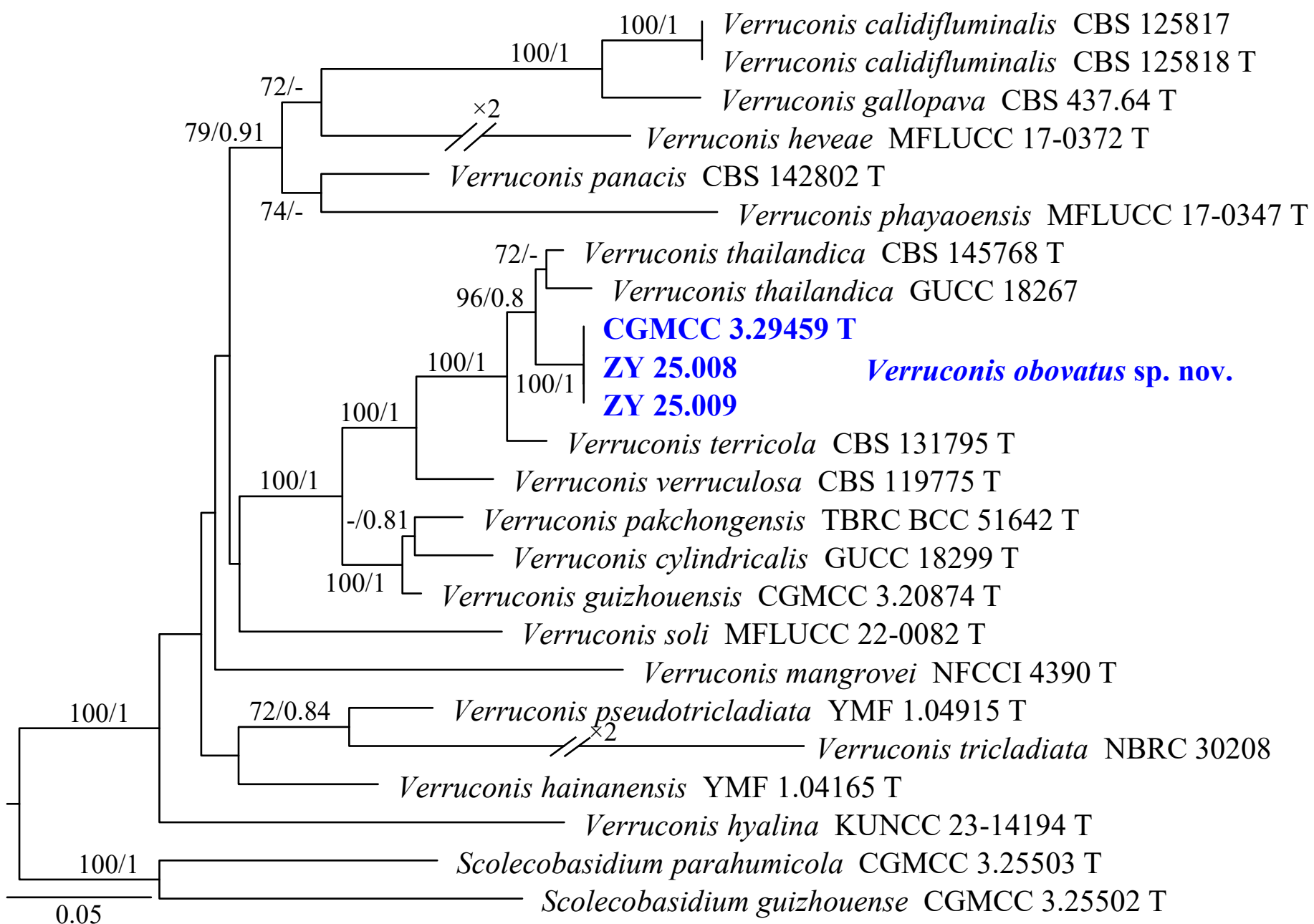

Supplement: Supplementary material 7 — Phylogenetic tree inferred from a maximum likelihood analysis based on a concatenated alignment of SSU, ITS, LSU, and tub2 sequences from 24 isolates representing the genus Verruconis and outgroup taxa [file mycokeys-133-209-s007.pdf]
